# Using Mathematical Algorithms to Modify Glomerular Filtration Rate Estimation Equations

**DOI:** 10.1371/journal.pone.0057852

**Published:** 2013-03-05

**Authors:** Xiaohua Pei, Wanyuan Yang, Shengnan Wang, Bei Zhu, Jianqing Wu, Jin Zhu, Weihong Zhao

**Affiliations:** 1 Division of Nephrology, Department of Geriatrics, The First Affiliated Hospital of Nanjing Medical University, Nanjing, Jiangsu, China; 2 Institute of Pattern Recognition and Machine Intelligence, School of Computer Science, Nanjing University of Science and Technology, Nanjing, Jiangsu, China; 3 Division of Respiration, Department of Geriatrics, The First Affiliated Hospital of Nanjing Medical University, Nanjing, Jiangsu, China; Mario Negri Institute for Pharmacological Research and Azienda Ospedaliera Ospedali Riuniti di Bergamo, Italy

## Abstract

**Background:**

The equations provide a rapid and low-cost method of evaluating glomerular filtration rate (GFR). Previous studies indicated that the Modification of Diet in Renal Disease (MDRD), Chronic Kidney Disease-Epidemiology (CKD-EPI) and MacIsaac equations need further modification for application in Chinese population. Thus, this study was designed to modify the three equations, and compare the diagnostic accuracy of the equations modified before and after.

**Methodology:**

With the use of ^99 m^Tc-DTPA renal dynamic imaging as the reference GFR (rGFR), the MDRD, CKD-EPI and MacIsaac equations were modified by two mathematical algorithms: the hill-climbing and the simulated-annealing algorithms.

**Results:**

A total of 703 Chinese subjects were recruited, with the average rGFR 77.14±25.93 ml/min. The entire modification process was based on a random sample of 80% of subjects in each GFR level as a training sample set, the rest of 20% of subjects as a validation sample set. After modification, the three equations performed significant improvement in slop, intercept, correlated coefficient, root mean square error (RMSE), total deviation index (TDI), and the proportion of estimated GFR (eGFR) within 10% and 30% deviation of rGFR (P_10_ and P_30_). Of the three modified equations, the modified CKD-EPI equation showed the best accuracy.

**Conclusions:**

Mathematical algorithms could be a considerable tool to modify the GFR equations. Accuracy of all the three modified equations was significantly improved in which the modified CKD-EPI equation could be the optimal one.

## Introduction

Chronic kidney disease (CKD) has evolved as a serious challenge to the health and well-being of world population [1], [2], among which China has not been spared. The latest incidence of CKD in China is 10.8% [3], equivalent to at least 100 million CKD patients.

Since the development of the Cockcroft-Gault equation in 1976, glomerular filtration rate (GFR) estimation equations have aroused global interests among nephrologists. Among a large number of variations, the Modification of Diet in Renal Disease (MDRD), Chronic Kidney Disease-Epidemiology (CKD-EPI) and MacIsaac equations have been publicly approved and applied [4]–[7]. However, ethnicity is one of the essential factors affecting accuracy of the GFR equations [8]. Previous validation studies indicated that modifications are indispensible for superior performances of the GFR equations in Chinese population [9]–[12].

Thus, the objective of this study was to create better GFR prediction models for Chinese population, with the first use of mathematical algorithms, due to their specialty at optimizing combinations.

## Methods

### Subjects

All participants in this study signed the informed consent. The participants with severe heart failure, acute renal failure, pleural or abdominal effusion, serious edema or malnutrition, skeletal muscle atrophy, amputation, ketoacidosis were excluded. Patients who recently received glucocorticoid and hemodialysis therapy were also excluded. Nanjing Medical University Ethics committee approved this study.

### Laboratory measurements

Serum creatinine (Scr) concentration was assayed by the enzymatic method (Shanghai Kehua Dongling Diagnostic Products Co., Ltd, China) with a reference range of 44∼136 µmol/L. Cystatin C concentration was examined by the particle-enhanced immunoturbidimetry method (Beijing Leadman Biomedical Co., Ltd, China) with a reference range of 0.60∼1.55 mg/L. Both two markers were examined by an Olympus AU5400 autoanalyzer (Olympus Co., Ltd, Japan).

### GFR measurement

A reference GFR (rGFR) was measured by ^99 m^Tc-DTPA renal dynamic imaging on a single photon emission computed tomography (Siemens E.CAM, Siemens Co., Ltd, Germany) [13]. Participants were informed in advance to have no special change in diet. After height and weight measurement, 300 ml water drinking, and bladder emptying, 185 MBq ^99 m^Tc-DTPA (purity 95%–99%, Nanjing Senke Co., Ltd, China) was injected into one of the veins of the participant. After images acquisition, rGFR was automatically calculated by a computer with the Gates method [14].

The estimation equations, including the MDRD, CKD-EPI and MacIsaac equations [15]–[17], were shown in Tables 1 and 2.

**Table 1:** Equations before and after modification by mathematical algorithms.

Name	Modification	Equation
MDRD	before	GFR = 186×Scr ^−1.154^×age^−0.203^ (×0.742, if female)
	after	GFR = 186×Scr ^−0.830^×age^−0.230^ (×0.742, if female)
MacIsaac	before	GFR = (86.7/cystatin C)−4.2
	after	GFR = (77.30/cystatin C)+2.32

MDRD: Modification of Diet in Renal Disease; GFR: glomerular filtration rate.

**Table 2:** The CKD-EPI equation before and after modification by mathematical algorithms.

Modification	Sex	Scr (µmol/L)	Equation
before	Female	≤62	GFR = 144×(Scr/0.7)^−0.329^×(0.993) ^age^
		≥62	GFR = 144×(Scr/0.7)^−1.209^×(0.993) ^age^
	Male	≤80	GFR = 141×(Scr/0.9)^−0.411^×(0.993) ^age^
		≥80	GFR = 141×(Scr/0.9)^−1.209^×(0.993) ^age^
after			
	Female	≤62	GFR = 144×(Scr/0.7)^0.156^×(0.993) ^age^
		≥62	GFR = 144×(Scr/0.7)^−1.057^×(0.993) ^age^
	Male	≤80	GFR = 141×(Scr/0.9)^0.074^×(0.993) ^age^
		≥80	GFR = 141×(Scr/0.9)^−1.057^×(0.993) ^age^

CKD-EPI: Chronic Kidney Disease Epidemiology Collaboration; GFR: glomerular filtration rate.

A single for the CKD-EPI equation is GFR = 141×min (Scr/κ, 1) ^α^×max (Scr/κ, 1)^−1.057^×0.993 ^age^ (×1.018, if female), where Scr is serum creatinine, κ is 0.7 for females and 0.9 for males, α is 0.156 for females and 0.074 for males, min indicates the minimum of Scr/κ or 1, and max indicates the maximum of Scr/κ or 1.

### Mathematical modification

The hill-climbing algorithm searched the local optimal solution by fixing each coefficient of the original equations, and orderly adjusting all the coefficients iteratively until no further improvement can be found. To avoid inaccuracy caused by various weights of coefficients, coefficient priorities were switched repeatedly. The simulated annealing algorithm searched the global optimal solution, which remedied the imperfection of the hill-climbing algorithm. Root mean square error (RMSE) was used to guide the modification process. Matlab software (version R2009a, Math Works Inc., USA) was the platform to accomplish the modification.

The entire modification process was based on a random sample of 80% of subjects in each GFR level as a training sample set, the rest of 20% of subjects as a validation sample set.

### Statistical Analysis

The Bias was calculated to show mean difference between eGFR and rGFR. Correlated coefficient was calculated using Pearson linear relation analysis to compare the correlation between various eGFR equations and rGFR. Slope and intercept were compared using Bland-Altman analysis. P_10_, P_30_ (the percentage of eGFR deviating within 10% and 30% of rGFR) [16]–[18] and the Total Deviation Index (TDI) [19] were also used to compare the accuracy of the equations before and after modification. *P* value less than 0.05 was taken to consider statistical significance. Statistical analyses were performed using SPSS software, version 16.0 (SPSS Inc., Chicago, USA) and Medcalc for Windows, (version 11.4.2.0, Medcalc Software, Mariekerke, Belgium).

## Results

A total of 703 Chinese subjects, including 422 males and 281 females, were recruited in this study, who attended The First Affiliated Hospital of Nanjing Medical University between December 2009 and October 2012. The subjects aged 18–95 yr, mean 52.38±16.86 yr, with the average rGFR, cystatin C and Scr 77.14±25.93 ml/min, 1.35±0.78 mg/L and 105.00±83.31 µmol/L, respectively. The GFR levels, medical history, detailed clinical characteristics of subjects were listed in Table 3.

**Table 3:** Detailed characteristics of 703 Chinese subjects.

Parameters	
**General characteristics**	Mean ± SD
Female	281 (40.0%)
Age (years)	52.38±16.86
Height (cm)	166.08±6.69
Weight (kg)	63.13±8.81
GFR (ml/min)	77.14±25.93
Cystatin C (mg/L)	1.35±0.78
Serum creatinine (µmol/L)	105.00±83.31
Blood urea nitrogen (mmol/L)	6.73±3.94
Uric acid (mmol/L)	353.40±124.93
Albumin (g/L)	42.84±22.04
Fasting blood glucose (mmol/L)	5.43±1.49
Low density lipoprotein (mmol/L)	3.25±0.75
**GFR levels**	Number of subjects (n)
GFR ≥90 ml/min	219 (31.2%)
GFR 60–89 ml/min	308 (43.8%)
GFR 30–59 ml/min	147 (20.9%)
GFR <30 ml/min	29 (4.1%)
**History of subjects**	Number of subjects (n)
Urinary tumor	176
Indeterminate etiology or unknown	106
Hematonosis	96
Urinary calculi	83
Hypertensive nephropathy	55
Obstructive nephropathy	53
Chronic nephritis	45
Diabetic kidney disease	41
Kidney donor	18
Renal tuberculosis	12
Simple renal cyst	7
ADPKD	6
Renal artery stenosis	5

GFR: glomerular filtration rate, measured by ^99 m^Tc-DTPA renal dynamic imaging; ADPKD: adult dominant polycystic kidney disease; values are means ± SD.

**Figure 1:**
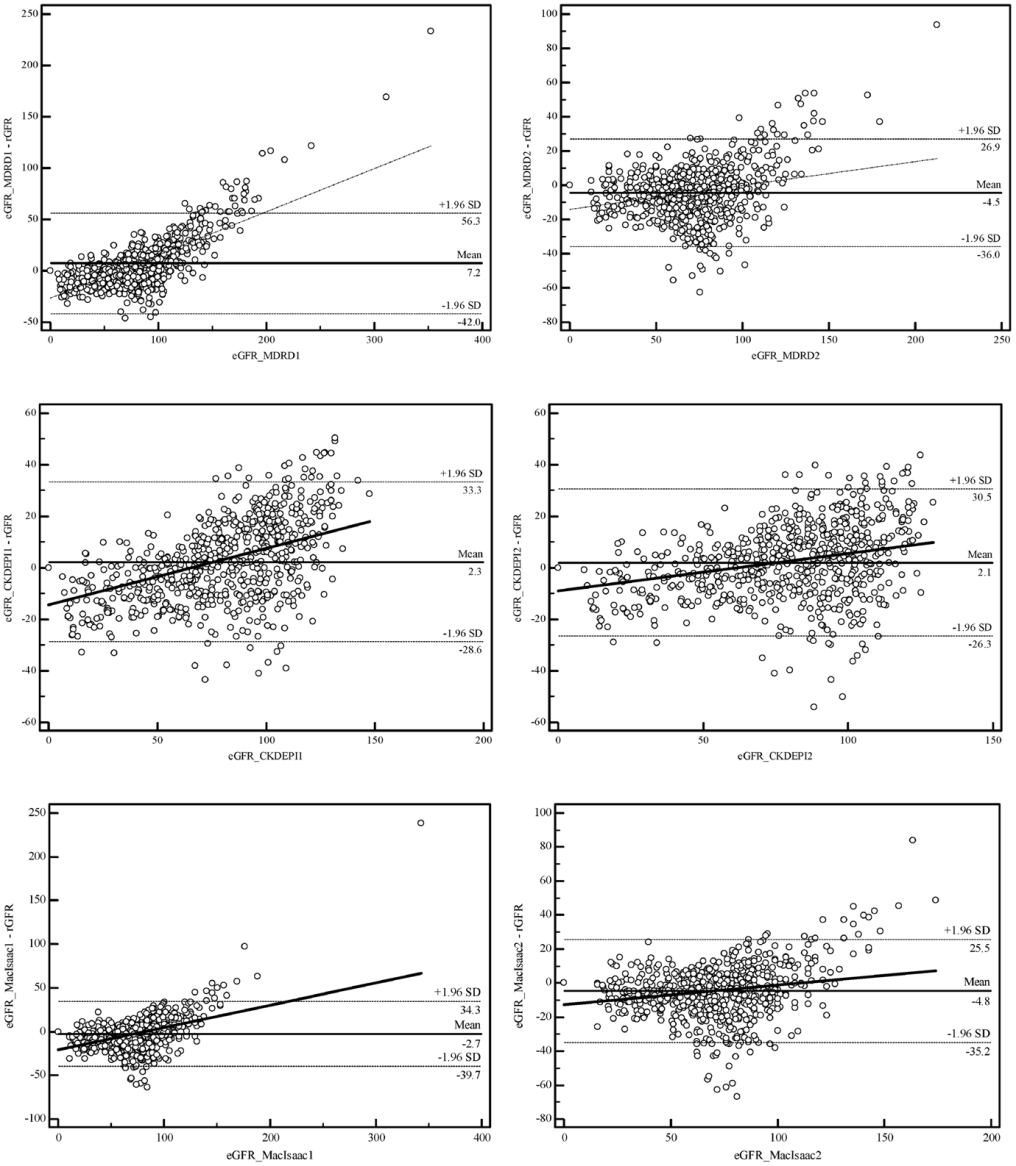
Comparison of agreement of the MDRD, CKD-EPI and MacIsaac equations before and after modification. MDRD: Modification of Diet in Renal Disease; CKD-EPI: Chronic Kidney Disease Epidemiology Collaboration; eGFR: estimated glomerular filtration rate; rGFR: reference glomerular filtration rate; eGFR_MDRD1: eGFR estimated by the original MDRD equation; eGFR_MDRD2: eGFR estimated by the modified MDRD equation. The oblique line represents the regression line of difference between eGFR and rGFR, showing slope and intercept. The solid horizontal line represents arithmetic mean between eGFR and rGFR, and the dotted line represents 95% confidence intervals of the standard deviation.

The modified equations were described in Tables 1 and 2. After modification, the trend to gather around rGFR turned prominent that the extremum or discrete data clearly reduced, and the correlation with rGFR tightened. The correlated coefficients of eGFR from the MDRD, CKD-EPI and MacIsaac equations rise from 0.784, 0.846 and 0.777 to 0.804, 0.851 and 0.810, respectively). Mean difference of the MDRD and CKD-EPI got smaller (MDRD: 7.42 ml/min decreased to −4.84 ml/min, CKD-EPI: 2.38 ml/min to 2.17 ml/min), but mean difference of the MacIsaac increased (−3.1 ml/min to −5.30 ml/min). Intercept and slope of eGFR from the MDRD, CKD-EPI and MacIsaac equations became narrowed in Bland-Altman analysis (intercept: −26.70, −14.26, −20.59 to 14.11, −8.91, −12.53, slope: 0.42, 0.22, 0.25 to 0.14, 0.15, 0.11, respectively) (Table 4, Fig. 1).

**Table 4:** Comparison of performance of glomerular filtration rate equations before and after modification in the validation database.

Assessment index	MDRD	CKD-EPI	MacIsaac
**Mean difference (ml/min)**			
Before	7.42	2.38	−3.10
After	−4.84	2.17	−5.30
**Slope**			
Before	0.42	0.22	0.25
After	0.14	0.15	0.11
**Intercept (ml/min)**			
Before	−26.70	−14.26	−20.59
After	−14.11	−8.91	−12.53
**Relationship**			
Before	0.784	0.846	0.777
After	0.804	0.851	0.810
**P_10_ (%)**			
Before	30.7%	32.3%	36.1%
After	35.7%	38.4%*	37.3%
**P_30_ (%)**			
Before	75.7%	79.4%	82.4%
After	84.1%**	84.5%*	85.3%
**Root mean square error**			
Before	0.321	0.256	0.233
After	0.227	0.227	0.225
**Total deviation index _70%_**			
Before	26.325	17.660	20.021
After	16.981	15.458	16.520
**Total deviation index _75%_**			
Before	30.974	20.778	23.557
After	19.980	18.187	19.438
**Total deviation index _80%_**			
Before	32.551	21.837	24.756
After	20.997	20.428	19.113

MDRD: modification of diet in renal disease; CKD-EPI: Chronic Kidney Disease Epidemiology Collaboration; Relationship: correlated coefficient of estimated GFR (eGFR) with reference GFR (rGFR); P_10_: proportion of eGFR that were within 10% of rGFR; P_30_: proportion of eGFR that were within 30% of rGFR.

* *P*<0.05,

** *P*<0.01, compared with the original equation.

Meanwhile, after modification, P_10_ of the MDRD, CKD-EPI and MacIsaac equations increased from 30.7%, 32.3%, 36.1% to 35.7%, 38.4%, 37.3%, synchronously, P_30_ increased from 75.7%, 79.4%, 82.4% to 84.1%, 84.5% and 85.3%. Another, TDI (including TDI 70%–80%) were also significantly decreased (Table 4).

**Figure 2:**
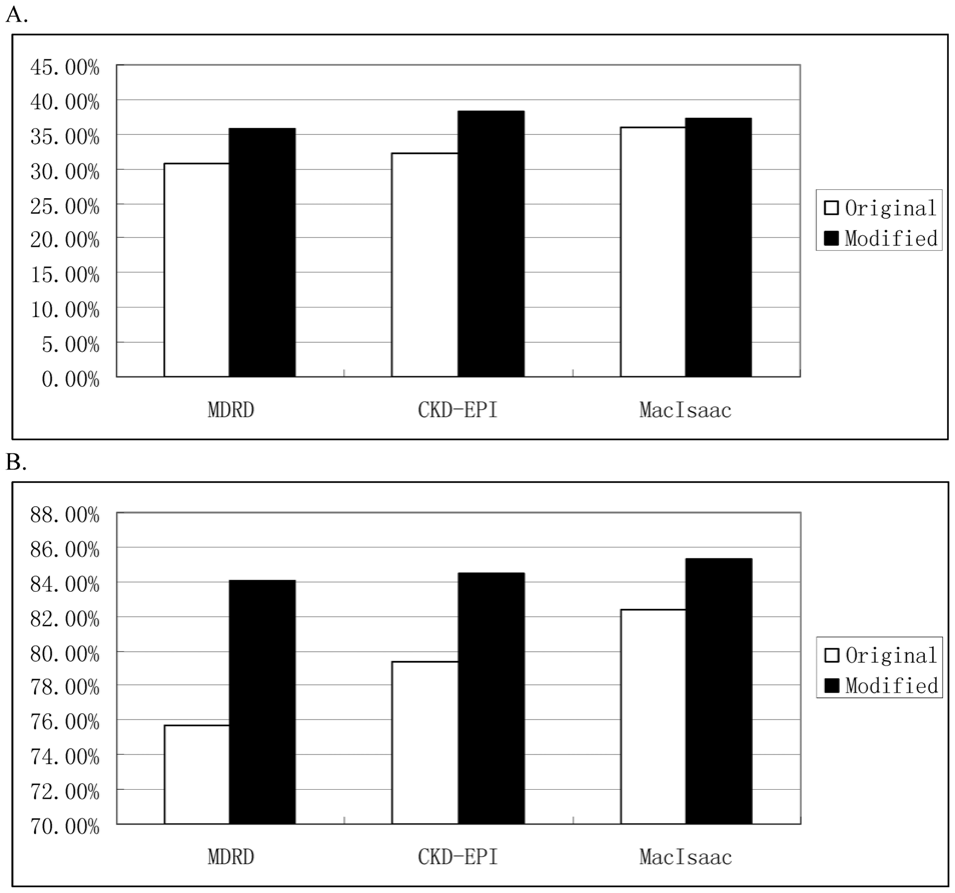
Comparison of accuracy of MDRD, CKD-EPI and MacIsaac equations before and after modification. A and B represent the percentage of eGFR deviating within 10% and 30% of rGFR, respectively. MDRD: Modification of Diet in Renal Disease; CKD-EPI: Chronic Kidney Disease Epidemiology Collaboration; eGFR: estimated glomerular filtration rate; rGFR: reference glomerular filtration rate; white column: original equation; black column: modified equation.

It was obvious that P_10_ and P_30_ of the modified Scr-equations increased (Table 4, Fig. 2). Compared with the modified cystatin C-based equation (MacIsaac equation), RMSE of the modified two Scr-based equations (MDRD and CKD-EPI equation) were decreased in sharp contrast (Table 4).

## Discussion

GFR is the core of CKD evaluation, diagnosis, and classification [20], [21]. Due to their simplicity, convenience, and low expense, the equations for GFR evaluation have been extensively applied worldwide [22], [23]. Especially, the MDRD and CKD-EPI equations have been successively recommended by Kidney Disease Outcomes Quality Initiative (K/DOQI) and Kidney Disease: Improving Global Outcomes (K/DIGO) [20], [24].

Since Scr is a classic biomarker of kidney function, most of the equations during the past 30 years were developed based on it. With the discovery of cystatin C [25], a potentially superior marker [26], [27], the equations based on it gradually created and popularized.

However, a great number of previous studies have consistently proved that “ethnicity” affects the accuracy of these equations, not only the Scr-based, but also the cystatin C-based [28]–[30]. Our precious studies [12], [31], [32] indicated that the CKD-EPI and MacIsaac equations could draw eGFR relatively closer to rGFR.

Due to the fact that China has the largest and fastest growing number of CKD patients in the world, it is of great significance to establish a more accurate GFR equation for Chinese population.

As powerful optimization capabilities of mathematical algorithms, we firstly introduced the mathematical algorithm to modify the present GFR estimation equations. According to the evidence above, the MDRD, CKD-EPI and MacIsaac equations finally were in selection to accept improvement.

The hill-climbing algorithm, a mathematical optimization technique of the local search family, was first introduced by Goldfeld in 1966 [33]. As an improvement of the depth-first search, the hill-climbing algorithm adopts heuristic strategy, which iteratively searches a better solution by orderly changing one coefficient to the next. However, the hill-climbing algorithm sometimes falls into the local optimization solution rather than the global optimization solution.

The simulated annealing algorithm is an anther artificial intelligence algorithm, which derived from the solid annealing principle. It was put forward by Metropolis in 1953 [34] and then applied into combinatorial optimization field by Kirkpatrick [35]. The simulated annealing algorithm has been widely used in fields such as very large scale integrated circuits, production scheduling, control engineering, machine learning, neural network, and signal processing [36]–[38]. The simulated annealing algorithm, based on iterative solution strategy, is a random optimization algorithm. The simulated annealing algorithm starts with a high initial temperature. Then it randomly searches the global optimization solution of the target function in the solution space with probabilistic jumping property, accompanied by the decline of the temperature parameter to compensate for the drawback of the hill-climbing algorithm.

In this study, the hill-climbing and simulated annealing algorithm substantially increased accuracy of the three selected equations. All the three modified equations performed significant improvement than the originals in slop, intercept, correlated coefficient, RMSE, P_10_, P_30_ and TDI. Of the three modified equations, the modified CKD-EPI equation showed the best accuracy.

It is interesting that after modification, improvement of RMSE, P_10_ and P_30_ in the Scr-based equations (MDRD equation and CKD-EPI equation) were more distinct than that of the cystatin C-based equation (MacIsaac equation). This fact indicated that Scr could be affected by ethnicity factor easier than cystatin C. Additionally, considering accuracy of the modified MacIsaac equation was similar to that of the modified CKD-EPI equation, plus its simple expression, the modified MacIsaac equation could be also recommended. Another matter should be stated that whether GFR should be adjusted for body surface area is still in debate and confused [39], [40]. Therefore, GFR in this study did not make the adjustment. In the end, owing to the inherent unequal distribution of the subjects in each GFR level, accuracy of the original GFR equations varied in different CKD stages [31]. Therefore, to minimize such bias, we modified the equations by stages. It is believed that the modified equations could be better suit for Chinese population.
